# Common Variable Immunodeficiency Associated with Hepatosplenic T-Cell Lymphoma Mimicking Juvenile Systemic Lupus Erythematosus

**DOI:** 10.1155/2011/428703

**Published:** 2011-06-08

**Authors:** A. A. Jesus, C. M. A. Jacob, C. A. Silva, M. Dorna, A. C. Pastorino, M. Carneiro-Sampaio

**Affiliations:** ^1^Rheumatology Division, Children's Hospital, Avenida Dr. Eneas Carvalho de Aguiar, 647, 05403-000 Sao Paulo, SP, Brazil; ^2^ Allergy and Immunology Division, Children's Hospital, Avenida Dr. Eneas Carvalho de Aguiar, 647, 05403-000 Sao Paulo, SP, Brazil; ^3^Division of Rheumatology, Hospital das Clínicas, Faculdade de Medicina, Universidade São Paulo, Av. Dr. Arnaldo 455, sala 3133, 01246-903 São Paulo, SP, Brazil

## Abstract

Common variable immunodeficiency (CVID) is a heterogeneous disorder with susceptibility to infections, autoimmune manifestations, and cancer. To our knowledge, CIVD with T-cell lymphoma mimicking juvenile systemic lupus erythematosus (JSLE) was not described in the literature, and one case was reported herein. An 8-year-old female was admitted in our Pediatric Immunology Unit with a clinical history of hypogammaglobulinemia, recurrent upper respiratory infections, and pneumonias. She had a marked decrease of three serum immunoglobulin isotypes, and the diagnosis of CVID was established. At the age of 17 years, she presented with oral ulceration, nonerosive arthritis, nephritis, serositis, cytopenia, positive antiphospholipid antibodies, and positive antinuclear antibody fulfilling the American College of Rheumatology (ACR) criteria for SLE. She was treated with intravenous methylprednisolone for three consecutive days, and intravenous immunoglobulin, and maintenance therapy of chloroquine, azathioprine and prednisone 40 mg/day. Two months later, she died of septic shock secondary to acute pneumonia. The necropsy showed hepatosplenic T-cell lymphoma with diffuse involvement of bone marrow, spleen, liver, and lungs. The lymphoma cells were positive for CD3 immunostaining and negative for CD20 and lysozyme. In conclusion, the association of CVID and hepatosplenic T-cell lymphoma may simulate JSLE diagnosis.

## 1. Introduction

The juvenile form of systemic lupus erythematosus (SLE) is a rare autoimmune disorder that may affect multiple organs and systems [1]. Of note, some primary immunodeficiencies (PIDs) are frequently associated to early-onset SLE or lupus manifestations, such as the deficiencies of the first components of the classical complement pathway and selective IgA deficiency [2]. On the other hand, PIDs with severe antibody synthesis deficiency, such as agammaglobulinemia and common variable immunodeficiency (CVID), have been rarely associated to SLE development [1].

CVID is a heterogeneous disorder with susceptibility to infections, autoimmune manifestations, and cancer [3] and has been classified as a predominant antibody deficiency according to the International Union of Immunological Societies (IUIS) updated classification [4]. This PID is characterized by a marked decrease of two serum immunoglobulin isotypes, usually IgG and IgM and/or IgA, over two standard deviations below mean values for age, in addition to impaired ability to specific antibody production after vaccination or exposure to a known infectious agent [3]. 

Autoimmune manifestations have been described in up to 20% of CVID patients [3]. The most common autoimmune complications reported are the cytopenias, especially immune thrombocytopenic purpura, and autoimmune hepatitis [3]. Additionally, systemic lupus erythematosus (SLE) was rarely reported in CVID patients [5], generally diagnosed during the disease followup. 

Furthermore, CVID patients have 2–8% of non-Hodgkins lymphoma, especially from B-cell origin [3]. However, to our knowledge, CVID with T-cell lymphoma mimicking juvenile SLE (JSLE) was not described in the literature, and one case was reported herein.

## 2. Case Report

An 8-year-old female was admitted to the Pediatric Immunology Unit with a clinical history of recurrent upper respiratory infections, pneumonias, and hypogammaglobulinemia. She presented with the first severe infection when she was 6 months old, needing hospitalization in intensive care unit (ICU). At 5 and 7 years old, she had two pneumonias with pleural effusion. On admission, aged 8 years old, physical examination detected weight and height on the 25th percentile. Laboratory exams demonstrated hemoglobin 12.5 g/L, hematocrit 40.1%, white blood cell count 6500 cells/mm^3^, platelets 211,000/mm^3^, and reduced serum levels of IgG 268–497 mg/dL (normal range 952–1538 mg/dL), IgA <6 mg/dL (normal 111–335), and IgM 55–122 mg/dL (normal 59–151). Specific IgG antibodies for measles and rubella were negative despite appropriate immunization. Lymphocyte immunophenotyping showed CD3^+^ 2085 cells/mm^3^ (normal 605 2460), CD4^+^ 936 cells/mm^3^ (normal 493–1666), CD8^+^ 937 cells/mm^3^ (normal 224–1112), CD16^+^/56^+^ 233 cells/mm^3^ (normal 73–654), and CD19^+^ 69 cells/mm^3^ (normal 72–520). Further flow cytometry tests showed CD19^+^ cells ranging from 0 to 4%. Therefore, CVID was diagnosed according to IUIS criteria (decrease of at least two serum immunoglobulin isotypes and negative specific antibody production after vaccination) [4], and prophylactic antibiotics and intravenous immunoglobulin (IVIG) were started. Antinuclear antibody (ANA) and rheumatoid factor (RF) were negative at that moment. The treatment resulted in the maintenance of IgG ≥600 mg/dL and in a reduced frequency of infectious episodes. However, during the followup, she was hospitalized eight times due to septic shock (*n* = 3), pneumonia with pleural effusion (*n* = 2), otomastoiditis (*n* = 1), acute cytomegalovirus infection (*n* = 1), and urinary tract infection (*n* = 1). At 12 years old, she developed pancytopenia [hemoglobin 10.2 g/L, hematocrit 34.2%, white blood cell count 3,790/mm^³^ (39% neutrophils, 54% lymphocytes, 2% eosinophils, and 5% monocytes), and platelets 108,000/mm^³^] associated to hepatosplenomegaly. Reticulocyte count was 1.2%, and lactate dehydrogenase (LDH) was 164 mg/dL (normal 117–213). Bone marrow aspiration was performed twice and showed hyperplasia of erythrocyte and hypoplasia of granulocyte series. At that moment, autoantibodies were not detected, such as: ANA, RF, antidouble-stranded DNA (anti-dsDNA), anti-Sm, anti-RNP, anti-Ro, anti-La, anti-P ribosomal, anticardiolipin IgG and IgM, lupus anticoagulant, anti-Scl70, anti-Jo1, anti-insulin, antineutrophil cytoplasmic (ANCA), antiglutamic acid decarboxylase (anti-GAD), antiinsulin, antithyroglobulin, antiperoxidase, antiparietal cell, antiendomysium, antismooth muscle, and anti-liver-kidney microsome antibodies. At the age of 17 years, the patient presented with fever, oral ulcers, alopecia, arthritis of wrists and elbows, headache, and cough and was hospitalized. She developed pleural and large pericardial effusion and was admitted to ICU. Laboratory exams revealed hemoglobin 7.9 g/L, hematocrit 22%, white blood cell count 1,000/mm^³^, platelets 17,000/mm^³^, reticulocyte count 0.32%, proteinuria 3.0 g/day, C3 72 mg/dL (normal 79 152), and C4 4 mg/dL (16–38). Polymerase chain reactions (PCR) for Epstein-Barr virus and blood and urine cultures were negative. The following autoantibodies were observed: ANA (1 : 320, dense fine speckled pattern), anticardiolipin IgM (100 MPL), RF, and lupus anticoagulant. At that moment, she fulfilled the American College of Rheumatology (ACR) [6] criteria for SLE. Additionally, she also had hepatosplenomegaly, aspartate aminotransferase (AST) 58 IU/l (normal 0–20 IU/l), alanine aminotransferase (ALT) 111 IU/l (normal 6–20 IU/L), triglycerides 456 mg/dL (normal <130 mg/dL), ferritin 6034 ng/mL (36–92 ng/mL), and LDH 791 mg/dL. She fulfilled the preliminary criteria for macrophage activation syndrome in pediatric SLE [7] and was treated with intravenous methylprednisolone for three consecutive days and IVIG (2 g/kg/dose) and, after chloroquine (250 mg/day), azathioprine (100 mg/day) and prednisone 40 mg/day. At that moment, bone marrow aspiration evidenced hypoplasia in all cell lineages without neoplastic cells or hemophagocytosis. Renal biopsy showed chronic tubulointerstitial nephritis and focal acute tubular necrosis without glomerular injury and negative immunofluorescence for IgA, IgG, IgM, C1q, C3, and fibrinogen, and liver biopsy showed drug-induced hepatitis without neoplastic cells. Despite treatment, two months later, she died of septic shock secondary to acute pneumonia. Remarkably, the necropsy showed hepatosplenic T-cell lymphoma (HSTL) with diffuse involvement of bone marrow, spleen, liver, and lungs. The lymphoma cells were positive for CD3 immunostaining and negative for CD20 and lysozyme.

## 3. Discussion

Lymphomas, especially from B-cell origin, have been described as associated to 2–8% of CIVD patients [3]. However, to our knowledge, this was the first case reported in the literature that described CVID with HSTL. Moreover, the clinical and laboratorial features mimicked JSLE.

Peripheral T-cell lymphomas are rare neoplasms. Two different subtypes are known: HSTL and primary cutaneous gammadelta T-cell lymphoma. Of note, HSTL is an extranodal lymphoma characterized by intrasinusoidal infiltration of the bone marrow, liver, and spleen [8], as observed in our patient. This entity has a rapidly and acute progressive course, with pancytopenia and lung involvement and without significant lymphadenopathy, as also evidenced herein [9]. This cancer commonly affects male adolescents and young adults [9] and may occur in patients under immune system suppression [8], particularly long-term immunosuppressive therapy for solid-organ transplantation [9]. Lymphocyte immunophenotyping is important for definitive diagnosis, especially expression of CD38. 

Importantly, CVID patients may present non-Hodgkin′s lymphoma generally in 6th and 7th decades and rarely in pediatric population. The most frequent subtype of this neoplasm is B cell without EBV infection [3]. Our female patient had a T-cell lymphoma in the second decade of life.

In addition, the patient described herein had JSLE clinical manifestations eleven years after CVID diagnosis. These findings contrast with the few case reports on the association between CVID and SLE that showed this primary immunodeficiency occurred during SLE followup. Fernández-Castro et al. [5] described two cases and reviewed the literature regarding SLE and CVID association. In all the 18 cases reported in the literature, CVID diagnosis was established one to 22 years after SLE diagnosis. In all these cases, hypogammaglobulinemia probably occurred as a complication of the immunosuppressive therapy [5]. To our knowledge, there is no case report of SLE after CVID or X-linked agammaglobulinemia diagnosis, and the autoantibody profile in this group of patients is unknown. Furthermore, it is unusual to see high titers of autoantibodies, as observed herein, in CVID-related autoimmunity [1]. 

Our patient had 7 of the 11 ACR classification 28 of lupus criteria (oral ulceration, nonerosive arthritis, proteinuria, serositis, hematologic abnormalities, positive antiphospholipid antibodies, and positive ANA) [6] and hypocomplementemia. Interestingly, the sensitivity and specificity of ACR 1982 criteria for pediatric lupus were described as 96% and 100%, respectively [10]. Despite this aspect, infectious diseases and malignancies should be always considered as differential diagnoses in pediatric lupus. Moreover, nephritis is frequent in pediatric lupus population, and renal biopsy did not show specific lupus nephritis findings with a surprisingly negative immunofluorescence in our case, indicating that the proteinuria was not related to lupus. The other clinical and laboratory features presented by the patient, including ANA and antiphospholipid antibodies, and initially attributed to lupus are nonspecific and could be related to neoplasm diseases. In addition, our patient did not show specific lupus autoantibodies (anti-dsDNA, anti-Sm, and anti-P ribosomal). Therefore, she probably had a lymphoma that mimicked lupus in a short period of time and not a neoplasm that complicated a preceding autoimmune disease. In fact, previously to the necropsy, various pediatric specialists worked up for this patient, and no malignancies were observed in the biopsies evaluated by expert pathologists of our teaching university hospital.

Lupus mimicking lymphoma in adult population was rarely described. Subcutaneous panniculitis-like T-cell lymphoma simulating a lupus erythematosus panniculitis with systemic manifestations was reported in one 29-year-old woman [11]. In contrast, JSLE with nephrotic syndrome and mediastinal lymphadenopathy mimicking lymphoma was described in one female adolescent [12].

Despite our patient had the preliminary criteria for macrophage activation syndrome with hyperferritinemia [7], these laboratory and clinical alterations are not specific for autoimmune diseases and can be found in lymphoma patients and infections [13].

In conclusion, the association of CVID and hepatosplenic T-cell lymphoma with diffuse involvement of bone marrow, spleen, liver, and lungs may simulate JSLE diagnosis.
